# Multimodality imaging with CT, MR and FDG-PET for radiotherapy target volume delineation in oropharyngeal squamous cell carcinoma

**DOI:** 10.1186/s12885-015-1867-8

**Published:** 2015-11-04

**Authors:** David Bird, Andrew F. Scarsbrook, Jonathan Sykes, Satiavani Ramasamy, Manil Subesinghe, Brendan Carey, Daniel J. Wilson, Neil Roberts, Gary McDermott, Ebru Karakaya, Evrim Bayman, Mehmet Sen, Richard Speight, Robin J.D. Prestwich

**Affiliations:** 1 Department of Radiotherapy Physics, St. James’ University Hospital, Leeds Teaching Hospitals NHS Trust, Leeds, UK; 2 Department of Nuclear Medicine, St. James’ University Hospital, Leeds Teaching Hospitals NHS Trust, Leeds, UK; 3 Department of Clinical Radiology, St. James’ University Hospital, Leeds Teaching Hospitals NHS Trust, Leeds, UK; 4 Department of Clinical Oncology, St. James’ University Hospital, Leeds Teaching Hospitals NHS Trust, Beckett Street, LS9 7TF Leeds, UK; 5 Department of Medical Physics, St. James’ University Hospital, Leeds Teaching Hospitals NHS Trust, Leeds, UK; 6 Department of Radiotherapy, St. James’ University Hospital, Leeds Teaching Hospitals NHS Trust, Leeds, UK

**Keywords:** Head and neck squamous cell cancer, Radiotherapy, Gross tumour volume, Delineation, Computed tomography, Fluorodeoxyglucose F18, Positron-emission tomography, Magnetic resonance imaging

## Abstract

**Background:**

This study aimed to quantify the variation in oropharyngeal squamous cell carcinoma gross tumour volume (GTV) delineation between CT, MR and FDG PET-CT imaging.

**Methods:**

A prospective, single centre, pilot study was undertaken where 11 patients with locally advanced oropharyngeal cancers (2 tonsil, 9 base of tongue primaries) underwent pre-treatment, contrast enhanced, FDG PET-CT and MR imaging, all performed in a radiotherapy treatment mask. CT, MR and CT-MR GTVs were contoured by 5 clinicians (2 radiologists and 3 radiation oncologists). A semi-automated segmentation algorithm was used to contour PET GTVs. Volume and positional analyses were undertaken, accounting for inter-observer variation, using linear mixed effects models and contour comparison metrics respectively.

**Results:**

Significant differences in mean GTV volume were found between CT (11.9 cm^3^) and CT-MR (14.1 cm^3^), *p* < 0.006, CT-MR and PET (9.5 cm^3^), *p* < 0.0009, and MR (12.7 cm^3^) and PET, *p* < 0.016. Substantial differences in GTV position were found between all modalities with the exception of CT-MR and MR GTVs. A mean of 64 %, 74 % and 77 % of the PET GTVs were included within the CT, MR and CT-MR GTVs respectively. A mean of 57 % of the MR GTVs were included within the CT GTV; conversely a mean of 63 % of the CT GTVs were included within the MR GTV. CT inter-observer variability was found to be significantly higher in terms of position and/or volume than both MR and CT-MR (*p* < 0.05). Significant differences in GTV volume were found between GTV volumes delineated by radiologists (9.7 cm^3^) and oncologists (14.6 cm^3^) for all modalities (*p* = 0.001).

**Conclusions:**

The use of different imaging modalities produced significantly different GTVs, with no single imaging technique encompassing all potential GTV regions. The use of MR reduced inter-observer variability. These data suggest delineation based on multimodality imaging has the potential to improve accuracy of GTV definition.

**Trial registration:**

ISRCTN Registry: ISRCTN34165059. Registered 2nd February 2015.

## Background

Target volume delineation in the treatment of head and neck cancers is a critical issue in the current era of highly conformal radiotherapy with intensity modulated radiotherapy (IMRT) techniques. Steep dose gradients allow sparing of adjacent critical structures but also introduce the potential for geographical misses leading to marginal recurrences if target volume delineation is not accurate [1–3]. Delineation variability can have a large impact on the dose to the tumour and organs at risk [4], and tumour delineation inaccuracy is recognised as a key source of error in radiotherapy delivery [5, 6]. Computed tomography (CT) remains the core of radiotherapy planning, with the electron density map generated providing accurate dosimetry. However, for delineation of the gross tumour volume (GTV) the limitations of CT-based delineation are widely acknowledged, and were clearly demonstrated in a study of the delineation of supra-glottic tumours with a 50 % degree of agreement between experienced physicians [7].

The integration of multimodality imaging into the radiotherapy planning process provides the opportunity to improve upon the reliance on CT-based tumour delineation. Magnetic resonance imaging (MR) offers excellent soft tissue discrimination, multiplanar imaging capabilities, and importantly, image quality is less susceptible to artefact from dental amalgam compared with CT [8, 9]. Anatomical imaging with CT or MR is inherently limited in allowing discrimination of tumour tissue from surrounding soft tissues. As a result, there has been considerable interest in utilising functional imaging to complement anatomical imaging [10, 11]. 2-Deoxy-2-[^18^F]-Fluoro-D-glucose positron emission tomography-computed tomography (FDG PET-CT) is a widely used functional imaging technique in oncology; tumour cells exhibit differential glucose uptake (the ‘Warburg effect’) as a basis of the identification of cancer [12]. The potential relevance of FDG PET-CT to radiotherapy planning is highlighted by the finding that loco-regional recurrences occur in-field in regions which are FDG-avid at baseline [13].

Some major institutions employ tight volumetric margins in the treatment of oropharyngeal cancer; for example recently reported series from major institutions [14–16] have employed GTV to CTV margins of 0-10 mm. However, the limited soft tissue contrast of CT commonly combined with interference from dental artefact make CT-based delineation of oropharyngeal primary tumours in routine clinical practice particularly challenging [17]. Therefore, the use of multimodality imaging to aid accurate GTV delineation for oropharyngeal primaries is appealing. However, only limited data is available to inform upon the intermodality comparison of CT, MRI and FDG PET-CT for oropharyngeal carcinoma [18, 19].

The primary aim of this prospective study was to quantitatively investigate the variation in oropharyngeal squamous cell carcinoma (OSCC) primary GTV delineation with CT, MR and FDG PET-CT, using volumetric and positional analyses.

## Methods

### Inclusion criteria

Inclusion criteria for this prospective single centre pilot imaging study were: age ≥18 years, histologically proven squamous cell carcinoma of the head and neck region, WHO performance status 0–2, decision to proceed with (chemo) radiotherapy with curative intent following discussion in a multi-disciplinary meeting, measurable primary cancer on routine pre-treatment imaging (CT and/or MR), and provision of fully informed consent. Patients were excluded from the study if there was poorly controlled diabetes, contraindication to MR or an estimated glomerular filtration rate <30 ml/min/1.73 m^2^. This study was approved by the Research Ethics Committee (National Research Ethics Committee Yorkshire and the Humber-Bradford, 11/YH/0212) and Administration of Radioactive Substances Advisory Committee (ARSAC); ISRCTN Registry: ISRCTN34165059 and all patients provided informed written consent prior to study entry.

The study protocol included contrast enhanced FDG PET-CT and MR scans performed in a 5-point thermoplastic radiotherapy immobilization mask. Target delineation and treatment proceeded according to institutional clinical protocols.

Fifteen patients entered the study; 1 patient withdrew consent prior to imaging. 11 of the 14 patients who underwent pre-treatment imaging according to the study protocol had a diagnosis of an oropharyngeal cancer and form the basis of this report.

### Image acquisition

#### FDG PET-CT

FDG PET-CT imaging was performed on a 64-section GE Discovery 690 PET-CT system (GE Healthcare, Amersham, UK). Baseline half-body PET acquisition and additional dedicated head and neck acquisition in the immobilization mask (3–4 bed positions, 2 minutes per bed position) from skull vertex to carina was performed for 60 minutes following a 400 MBq injection of Fluorine-18 FDG intravenously. The CT component of the head and neck acquisition was obtained after a 25 second delay following a bolus of 100 ml of iodinated contrast (Niopam 300, Bracco Ltd, High Wycombe, UK) injected at 3 ml/s using the following settings; 120 kV, variable mA (min 10, max 600, noise index 12.2), tube rotation 0.5 s per rotation, pitch 0.969 with a 2.5 mm slice reconstruction. The head and neck component of the FDG PET-CT scan, acquired with a 5-point thermoplastic radiotherapy immobilization mask fitted and room laser alignment to radiopaque reference markers placed on the mask, was also used for radiotherapy planning according to routine clinical protocols.

#### MR

MR images were acquired on a 1.5 T Siemens Magnetom Avanto system (Siemens Healthcare, Erlangen, Germany). Patients were immobilized in the same treatment position and the same mask as for FDG PET-CT imaging. Axial post-contrast T1-weighted (TR = 831 ms, TE = 8.6 ms, 105 × 2 mm thick contiguous slices, acquired voxel size = 0.9 × 0.9 × 2.0 mm) and axial fat saturated T2-weighted (TR = 4430 ms, TE = 76 ms, voxel size = 0.8 × 0.7 × 3.0 mm) sequences were acquired.

### Image co-registration

To allow the spatial comparison of the FDG PET-CT, CT and MR scans, rigid image registration was undertaken using Mirada RTx v1.4 software (Mirada Medical, Oxford, UK) between the CT dataset and the T1-weighted MR dataset. FDG PET-CT scans were inherently spatially co-registered.

### Gross tumour volume delineation of primary tumour

In order to simulate the clinical scenario, all outlining was performed with access to clinical history, findings of clinical examination, diagnostic imaging including CT and/or MR performed as part of the diagnostic process prior to entry into the study; FDG PET-CT was not performed as a routine diagnostic investigation and was not therefore available to the observers.

### CT and MR based GTV contours

For each patient, five observers (two radiologists and three radiation oncologists) were provided with lists of contours to be performed on study images of primary tumours (CT, MR and combined CT and MR (CT-MR)); the order in which contours were performed was systematically varied for each individual observer. To minimize any potential for recall, a minimum of a two week interval was mandated prior to generating contours for each individual patient using different imaging modalities. For CT based contours, observers were blinded to the MR and PET images acquired as part of the study protocol. For MR based contours, post-contrast T1-weighted and fat saturated T2-weighted images were available and inherently co-registered; and observers were blinded to CT and PET images acquired as part of the study protocol. For combined CT-MR contours, the post-contrast T1-weighted and fat saturated T2-weighted MR and CT were available.

### FD***G*** PET-CT GTV contours

Image analysis was undertaken on Mirada RTx v1.4 software. The maximum standardized uptake value (SUV_max_) was derived by drawing a region of interest (ROI) encompassing the primary tumour. The PET GTV was defined by using an adaptive thresholding technique, known as the Schaefer algorithm [20], calculated from the mean primary tumour SUV (SUV_mean_) when applying a 70 % of SUV_max_ isocontour, the background tissue SUV_mean_ and two scanner specific coefficients (determined from phantom studies).

### Data analysis

The data analysis was split into the GTV volume analysis and position analysis. All statistical analysis was performed using Matlab^2013b^ (MATLAB and Statistics Toolbox Release 2013b, The MathWorks, Inc., Natick, Massachusetts, United States).

### Volume analysis

#### Variation in volume of GTV with modality

Linear mixed effects models were used to determine the significance of differences in GTV volume with modality, where modality and clinician role (radiologist or radiation oncologist) were fixed effect variables and patient and clinician were random effect variables [21]. The lack of multiple clinician PET GTVs made inter-clinician variability impractical to model when PET was included, therefore multiple models were used where clinician and clinician title inter-observer variability terms were excluded in the PET GTV model. Data population testing was performed using Q-Q plots and × ^1/3^ transformations were used to create normal population distributions. A significant *ρ*-value was considered to be *ρ* < 0.02 to account for the multiple model comparisons that were required due to the fixed variable comparison method in linear mixed effects models [22].

#### Variation in volume of GTV with clinician group

The mean GTV volumes for the CT, MR and CT-MR modalities were calculated for each clinician group; radiologist and oncologist. Significance testing between clinician groups for each modality was undertaken using linear mixed effects models.

#### Variation in inter-observer variability with imaging modality

The variation in inter-observer delineation was measured by taking the mean over all patients of the standard deviation of all observers delineations for each patient within a modality. This was repeated for CT, MR and CT-MR volumes. Significance testing was then performed between modalities using an ANOVA test combined with a Tukey multiple comparison test [23].

### Positional analysis

Six positional metrics were calculated using ImSimQA software (v3.1.5, OSL, Shrewsbury, UK): Mean distance to conformity (MDC); Centre of gravity distance (CGD); Conformity index (CI); DICE index; sensitivity index (Se. Idx); and inclusion index (Incl. Idx). The conformity index and DICE index both produce output values between 0 and 1 (using different calculation methods), where 0 represents two contours with no overlap and 1 represents two contours that are perfectly overlapping [24]. The Se. Idx and Incl. Idx calculate the overlapping volume between two contours as a percentage of the volume of one of the two contours. When used together they calculate the percentage of volume A which is within volume B and vice versa. CGD is the distance between the geometric centres of two contours [25]. MDC is the mean of the distances between contours averaged over all positions not within the overlapping contour [25].

### Variation in inter-observer variability with imaging modality

The positional inter-observer variability for each modality was assessed by comparing all GTVs delineated using the same modality for each patient. The final positional comparison values were calculated for each metric by calculating the mean of the metric results for each patient and subsequently the overall mean result for all patients. Significance testing was then performed between modalities using an ANOVA test combined with a Tukey multiple comparison test [23].

### Variation in GTV position with imaging modality

The variation in GTV position between modalities was assessed using ImSimQA between GTVs delineated by the same clinician and the PET GTV for each patient.

## Results

**Fig. 1:**
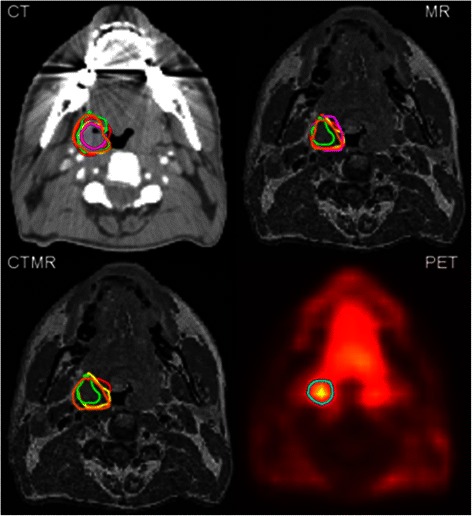
Example of inter-observer variability in contouring GTVs based on CT, MR, CT-MR and of auto-segmented contour based on PET for a patient with a T2 N2b poorly differentiated squamous cell carcinoma of the right tonsil. Contours shown are: radiation oncologist 1 red, radiation oncologist 2 yellow, radiation oncologist 3 orange, radiologist 1 green, radiologist 2 purple, PET contour blue

**Fig. 2:**
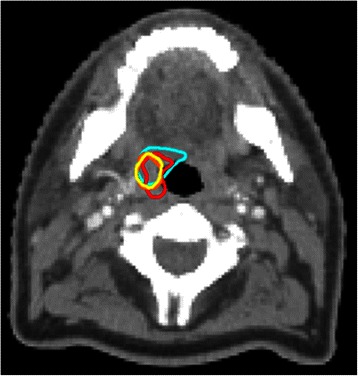
Representative example of GTVs delineated on CT, MR, CT-MR by a single radiation oncologist, displayed on an axial CT scan, for a patient with a T1 N2b well differentiated squamous cell carcinoma of the right tonsil. CT GTV red; MR GTV yellow; CT-MR contour orange; PET contour blue

11 patients with histologically proven OSCC entered the study. Baseline characteristics are summarised in Table 1. Diagnostic imaging included MR for all patients. The median time between FDG PET-CT and MR scans performed within the study was 7 days (range 0–12). Within the time constraints for completing contouring of the primary tumour GTV, all CT contours, 51/55 MR, and 42/55 CT-MR GTV contours were completed; 10/11 combined CT-MR GTVs were incomplete for one radiologist. A representative example of contours delineated by each observer on CT, MR, CT-MR and by automatic segmentation of PET is shown in Fig. 1. Figure 2 provides an example of contouring by a single observer on CT, MR, CT-MR and by automatic segmentation of PET superimposed upon the CT scan.

**Table 1:** Patient demographics and tumour characteristics

Patient	Primary Tumor Site	T-stage	N-stage
1	Tonsil	2	2b
2	Base of tongue	3	2c
3	Base of tongue	4	2b
4	Base of tongue	4a	1
5	Base of tongue	2	1
6	Tonsil/base of tongue	1	2b
7	Base of tongue	2	2b
8	Base of tongue	2	2b
9	Tonsil/soft palate	4a	1
10	Base of tongue	1	2b
11	Soft palate	4a	2b

### Volume analysis of GTVs

**Fig. 3:**
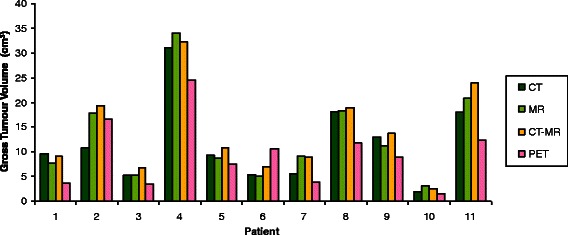
Median volumes of GTVs delineated on CT, MR, CT-MR and PET for each patient

**Fig. 4:**
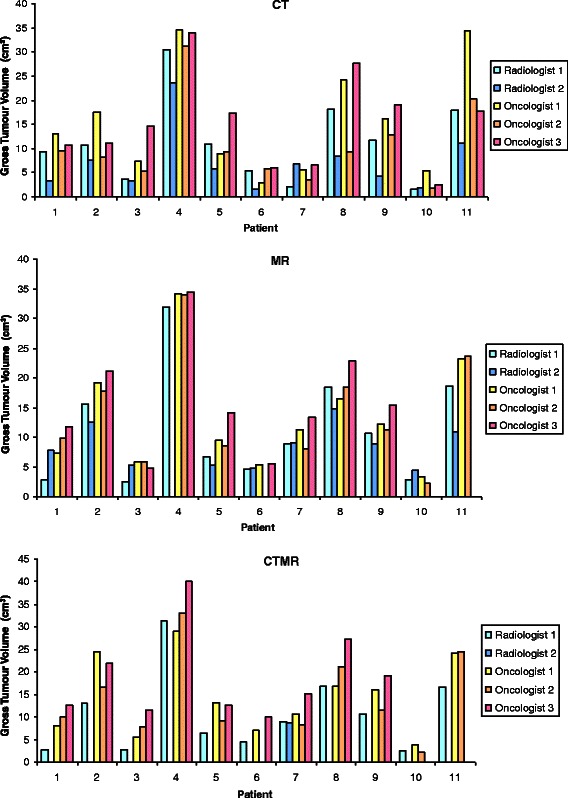
Volumes of GTVs delineated by individual observers on CT, MR and CT-MR

The volume of the primary tumour contours for CT, MR, CT-MR and PET are shown for each patient in Fig. 3 and are summarised in Table 2. Table 2 illustrates the median and mean volumes of GTVs delineated on CT, MR, CT-MR and generated by automatic segmentation of the PET. Figure 4 demonstrates the volume of GTVs delineated by individual observers using CT, MR and CT-MR. Table 3 illustrates the standard deviation of the GTV volume delineations for each patient for each modality. Compared with CT GTVs, CT-MR GTVs were significantly larger (*p* = 0.0052). MR had a significantly smaller GTV volume standard deviation than CT (*ρ*-value < 0.05). Average PET GTVs were smaller than CT, MR and CT-MR volumes, a difference which was significant compared with MR and CT-MR GTVs (*p* = 0.003 and *p* < 0.001 respectively).

**Table 2:** Summary of volume of GTVs contoured using CT, MR, CT-MR and PET

Modality	Modality Volumes (cm^3^)					
	Mean	Median	Mean St. Dev.	Range		Mean GTV Volume (Statistically Significant *p*-values)
				Max	Min	
CT	11.9	11.6	4.5	34.5	1.6	CT < CT-MR, *p* = 0.005
CT-MR	14.1	14.0	3.7	40.2	2.2	CT < MR, *p* = 0.049
MR	12.7	12.8	2.5	34.4	2.2	MR < CT-MR, *p* = 0.33
PET	9.5	8.8	-	24.6	1.5	PET < CT, *p* = 0.059
						PET < CT-MR, *ρ* <0.001PET < MR, ρ = 0.003

**Table 3:** The standard deviation of the GTV volume delineations undertaken by clinicians for each patient for each modality

Patient	Standard Deviations (cm^3^)		
	CT	CT-MR	MR
1	3.59	4.17	3.33
2	3.95	5.19	3.32
3	4.61	3.75	1.36
4	4.36	4.82	1.15
5	4.29	3.16	3.37
6	2.01	2.81	0.48
7	1.84	2.84	2.15
8	8.65	4.87	2.99
9	5.63	3.91	2.40
10	1.62	0.83	0.93
11	8.59	4.42	5.93
Mean	4.47	3.71	2.49

Significant differences were found between radiologist- and oncologist-delineated GTV volumes for each individual modality: CT (radiologist 9.1 cm^3^ vs. oncologist 13.8 cm^3^, *ρ* = 0.022); MR (radiologist 9.9 cm^3^ vs. oncologist 14.4 cm^3^, *ρ* = 0.00013); CT-MR (radiologist 10.5 cm^3^ vs. oncologist 15.8 cm^3^, ρ = 0.12); and overall for all modalities (radiologist 9.7 cm^3^ vs. oncologist 14.6 cm^3^, *ρ* = 0.001).

### Positional analysis of GTVs

The analysis of positional inter-observer variability is summarized in Table 4. Inter-observer variability was found to be significantly higher for CT compared to MR and CT-MR, with no significant differences between MR and CT-MR contours.

**Table 4:** Mean positional metric results for the inter-observer variability

	Mean Inter-observer Variability (SD)			Significant Differences with a confidence level of 95 %
	CT	CT-MR	MR	(*p*-value <0.05)
CI	0.37 (0.12)	0.44 (0.09)	0.47 (0.09)	CT < CT-MR
				CT < MR
MDC (mm)	8.8 (4.1)	7.5 (2.6)	6.9 (2.5)	MR < CT
CGD (mm)	7.7 (4.5)	4.8 (2.6)	4.4 (2.5)	CT-MR < CT
				MR < CT
DICE	0.57 (0.15)	0.66 (0.09)	0.69 (0.10)	CT < CT-MR
				CT < MR

The results of the comparison of GTV position between CT, MR, CT-MR and PET is shown in Table 5. CT, MR and CT-MR were found to all have similar, large differences in position compared to PET. A mean of 64 %, 74 % and 77 % of the PET GTV were included within the CT, MR and CT-MR GTVs respectively. A mean of 56 %, 58 %, 54 % of the CT, MR and CT-MR GTVs were included within the PET GTVs. MR and CT GTVs were found to have a low level of overlap and a large variation in CGD and MDC. A mean of 57 % of the MR GTV was included within the CT GTV; conversely a mean of 63 % of the CT GTV was included within the MR GTV. MR and CT-MR were found to have a high level of overlap and a small variation in CGD and MDC; a mean of 85 % of the CT-MR GTV was included within the MR GTV .

**Table 5:** Inter-modality positional GTV analysis

Metric	Inter-Modality Variability (SD)					
	CT-PET	MR-PET	CTMR-PET	CT-MR	CT-CTMR	MR-CTMR
CI	0.33 (0.09)	0.36 (0.05)	0.36 (0.07)	0.35 (0.09)	0.40 (0.08)	0.74 (0.17)
MDC (mm)	7.8 (3.1)	6.9 (2.4)	7.3 (2.87)	7.1 (1.7)	6.7 (1.6)	4.1 (0.9)
CGD (mm)	6.1 (3.2)	4.7 (2.2)	4.9 (3.64)	5.9 (3.1)	5.4 (2.7)	1.8 (1.4)
DICE	0.55 (0.11)	0.61 (0.06)	0.60 (0.08)	0.57 (0.09)	0.62 (0.09)	0.87 (0.10)
Se. Idx^a^	0.56 (0.15)	0.58 (0.11)	0.54 (0.13)	0.63 (0.14)	0.67 (0.14)	0.91 (0.09)
Incl. Idx^b^	0.64 (0.18)	0.74 (0.11)	0.77 (0.13)	0.57 (0.14)	0.63 (0.14)	0.85 (0.13)

^a^Se. Idx is expressed as a proportion of the first named GTV contained within the second ie. for CT-PET Se. Idx is the proportion of the CT-GTV within the PET-GTV

^b^Incl. Idx is expressed as a proportion of the second named GTV contained within the first ie. for CT-PET Incl. Idx is the proportion of the PET-GTV within the CT-GTV

## Discussion

There is considerable interest in improving the accuracy of tumour delineation in the era of highly conformal IMRT [10]. The current standard of CT-based delineation is particularly limited for oropharyngeal primary tumours, which are often barely visible even with contrast-enhanced CT-simulation scans [9, 19]. Multimodality imaging has the potential to improve the accuracy and reproducibility of tumour delineation.

Clinical experience suggests that oropharyngeal primary tumours are more readily identifiable on MR than CT. There was no significant difference in the volume of GTVs outlined on MR and CT. Although there was considerable inter-observer variability for CT, MR and CT-MR GTV delineation, there was significantly less variability for MR and CT-MR than for CT GTVs. Analysis of positional metrics demonstrated a low degree of volume overlap between CT and MR GTVs. MR and CT-MR GTVs showed a large degree of overlap; this is likely to reflect the clinicians’ propensity to base the CT-MR GTV contours on the MR on which the edge of the primary tumour is more readily identifiable. These data suggest that the implementation of either combined CT-MR or MR-based planning would have a considerable impact upon GTV delineation compared with CT-based planning.

These data are broadly in line with a previous study by Daisne et al. [18] who did not find a significant difference in the volume of GTVs contoured by a single observer on CT or MR in a series of 10 patients with oropharyngeal carcinoma. Consistent with our results, this series also showed significant areas of non-overlap between CT and MR defined GTVs. Another prior study by Ahmed et al. compared CT and MR-based GTVs in a series of six patients with base of tongue cancers [17]. This study also found that there was only limited overlap between CT and MR GTVs although, by contrast with our results, reported that there was no difference in inter-observer variability between CT and MR and that the primary tumour GTV was larger on MR than CT.

Interestingly our data showed that GTVs delineated on CT, MR or CT-MR were significantly smaller when contoured by radiologists compared with oncologists. Similarly, Ahmed et al. [17] reported that average GTVs delineated by a single radiologist were smaller than those contoured by oncologists. Clinical information and the findings of clinical examination remain critical to avoid geometric misses due to disease such as mucosal extension which may not be identified on imaging. Variations in this study between oncologists and radiologists emphasize the potential benefit of a multidisciplinary collaborative approach to GTV delineation, including radiation oncologists, radiologists and surgeons (who may have valuable additional input, for example based on the findings of an examination under anaesthetic).

With regard to the use of FDG PET-CT for radiotherapy planning, a key issue is the methodology used to define the edge of the functional volume of interest. Current generation PET-CT scanners have limitations including image noise, voxel sizes of 4-5 mm, partial volume effects and reconstruction uncertainties which lead to blurring of the edge of PET-avid tumours [9]. A host of methods have been proposed for ‘contouring’ a PET-avid tumour, varying from manual visual delineation to fully automated algorithms [26, 27]. Altering the SUV scale when viewing PET images can alter the apparent tumour volume by a factor of around two [28]; manual delineation is therefore an inevitably subjective process leading to inter-observer variability [29]. Although a host of automated methods have been developed for segmenting PET-avid tumours [30], few have histopathological correlation. In the absence of a widely accepted method, we made a pragmatic decision to use a previously described contrast-orientated method with coefficients derived from individual phantom data on the PET-CT scanner which had performed favourably in comparative phantom and simulated patient studies [20, 31, 32], and pathological correlation in other tumour sites [33]. The results from the PET delineation component of this study need to be interpreted with the unresolved difficulty regarding the optimal method of PET delineation in mind.

PET-based GTVs were significantly smaller than MR and CT-MR GTVs (Table 2), and non-significantly smaller than CT GTVs. Despite this difference in volume, there were substantial areas of the PET GTV which were not included in the CT or MR GTVs; conversely large areas of the CT and MR GTVs were not included within the PET GTV. Consistent with these findings, was the reported series of Daisne et al. [18] of 10 patients with oropharyngeal cancer in which the PET GTV was significantly smaller than CT or MR-based GTVs, with areas of mismatch between PET GTVs and CT or MR GTVs. Interestingly, for patient 6 the PET GTV volume was greater than any other modality GTV volume. This was in contrast to all other patients and the overall results of this study. This could be due to the inherent difficulties in delineating a PET GTV that occur, even using the semi-automatic contouring algorithm, when the GTV ^18−^FDG uptake resides in an area of natural ^18−^FDG uptake caused by, for example, inflammation or brown fat. In such cases the PET GTV delineation can incorrectly identify physiological ^18−^FDG uptake as tumour uptake, leading to false positive GTV tissue and a larger GTV delineation than appropriate. In this case, when visually reviewed it was found that the PET GTV extended further inferiorly compared to the other modality GTVs and also was in a region of relatively high background uptake around the tonsils.

The main limitation of this series is the absence of histological validation. Two series including nine [18] and ten [34] patients who underwent a laryngectomy/laryngopharyngectomy for laryngeal or hypopharyngeal cancer following CT, MR and FDG PET-CT imaging have provided histological validation. Both series reported that the pathological tumour was smaller than any individual imaging modality, but that no single imaging modality encompassed the whole pathological tumour. The inability of imaging to depict the whole tumour volume was thought likely to be due to superficial mucosal extension in that tumour site. No similar series with pathological correlation have been performed for oropharynx cancers to the best of our knowledge. By contrast with the larynx, a resected specimen from the oropharynx would lack the cartilage structure to provide registration with imaging; in addition, oropharyngeal cancer is commonly managed non-surgically. In the absence of pathological validation, our series is descriptive without a ground truth; it is important to recognise that increasing the consistency of contours does not necessarily imply superior target volume delineation. Other limitations include the necessity for co-registration between MR and FDG PET-CT scans; since both scans were performed within the same immobilisation mask it would be expected that co-registration errors would be small.

In the absence of histological validation, it is not possible to select which imaging modality is superior for target volume delineation. It is perhaps not surprising that anatomical and functional imaging techniques provide potentially complimentary information. The smaller FDG-PET volume may be demonstrating the inability of the other techniques to discriminate between inactive necrotic/cystic tissue and the active cancerous tissue; however, FDG uptake is non-specific, so areas of FDG uptake beyond CT or MRI-delineated tumour volume may relate to adjacent inflammatory changes or alternatively areas of sub-clinical tumour infiltration. It seems likely that incorporating multimodality imaging with accurate clinical examination will minimise the risk of a geographical miss. For example, PET may add to the accuracy of target delineation based on anatomical imaging by the detection of areas which are FDG-avid but sub-clinical on CT and MR. This is supported by the findings of Thiagarajan et al. [19] who reported on the impact of PET and MR and physical examination in target delineation in a series of 41 patients with oropharyngeal cancer. This study compared a reference GTV based on CT, PET, MR and physical examination; the concordance indices for both GTVs based on CT and MR or based on CT and PET were low compared with the reference GTV, implying a potential benefit for incorporating all imaging modalities. Importantly, the study highlighted the importance of clinical examination in addition to multimodality imaging for the detection of mucosal extension.

These data show the potential complimentary role for multimodality imaging in target volume delineation. Clearly additional multicentre prospective clinical studies are needed to analyse the impact of this approach on clinical outcomes. Incorporation of multimodality imaging may be more beneficial in the advanced disease setting (patients in this study all had stage III/IV disease) compared with the treatment of early disease. The impact of multimodality imaging on the balance of achieving local control whilst minimising toxicity will depend upon the approach and margins adopted to delineating the clinical target volume, as a multimodality imaging-defined GTV may be larger than that defined on CTV alone. A cost-effectiveness analysis will be useful prior to widespread incorporation into routine practice.

## Conclusion

In summary, this study showed that using CT, MR and PET produced significantly different GTVs which varied in volume and/or position, with no single imaging modality encompassing all potential GTV regions. These data support the increased incorporation of multimodality imaging for target volume delineation, to minimise the risk of geographical misses.

## Abbreviations

ARSAC: administration of radioactive substances advisory committee
CGD: centre of gravity distance
CI: conformity index
CT: computed tomography
CTV: clinical target volume
FDG: 2-deoxy-2-[^18^F]-fluoro-D-glucose
GTV: gross tumour volume
IMRT: intensity modulated radiotherapy
Incl. Idx: inclusion index
MDC: mean distance to conformity
MR: magnetic resonance imaging
OSCC: oropharyngeal squamous cell cancer
PET: positron emission tomography
ROI: region of interest
Se Idx: sensitivity index
SUV_max_: maximum standardized uptake value
